# New Rectum Bougie

**Published:** 1868-10

**Authors:** J. S. Sherman

**Affiliations:** Chicago


ARTICLE XLII.

### NEW RECTUM BOUGIE.

BY J. S. SHERMAN, M.D., Chicago.

A bougie is often required which can be passed up the rectum into the sigmoid flexure. The ordinary rectal bougies are too stiff to accommodate themselves to the course of the bowel, while the more flexible double upon themselves before sufficient pressure is used to open the upper sphincter. A bougie should be flexible enough to bend to the hollow of the sacrum and curve of the bowel, and at the same time sufficiently rigid to force its way without coiling.

Lead is a substance possessing such properties, and from this metal I have constructed a bougie in the following manner:

Have a lead rod drawn out the required length, and a little less than one-third of an inch in diameter. This is introduced into an elastic catheter, or piece of rubber tubing. This covering is necessary, as lead, from frequent bending, is liable to break; and, if not thus enclosed, there would be danger of losing portions in the rectum.

A piece of hard rubber or wood, turned in an oval shape, and attached to the bougie with a rivet, completes the instrument. Various sizes of these may be turned, and used on the same lead rod. For exploring the rectum high up, such an instrument will be found very useful. It may be made hollow, and used for injecting the bowel in cases of intussusception.

